# Psoriasis under B-cell depleting therapies in multiple sclerosis: a retrospective multicenter analysis

**DOI:** 10.1177/17562864261427169

**Published:** 2026-03-19

**Authors:** Patricia Kirschner, Franz F. Konen, Franziska Axhausen, Ulas Ceylan, Erda Bucak, Romy Baumgart, Lars Masanneck, Stefan Gingele, Kerstin Steinbrink, Sven G. Meuth, Ralf Gold, Stephanie Wolff, Simon Faissner, Thomas Skripuletz, Steffen Pfeuffer, Marc Pawlitzki

**Affiliations:** Department of Neurology, Medical Faculty, Heinrich-Heine-University Düsseldorf, Moorenstraße 5, Düsseldorf 40225, Germany; Department of Neurology, Hannover Medical School, Hanover, Germany; Department of Neurology, Justus-Liebig-University Giessen, Giessen, Germany; Department of Neurology, St. Josef-Hospital, Ruhr-University Bochum, Bochum, Germany; Department of Neurology, Hannover Medical School, Hanover, Germany; Department of Neurology, Justus-Liebig-University Giessen, Giessen, Germany; Department of Neurology, Medical Faculty, Heinrich-Heine-University Düsseldorf, Düsseldorf, Germany; Department of Neurology, Hannover Medical School, Hanover, Germany; Department of Dermatology, University Hospital Münster, University of Münster, Münster, Germany; Department of Neurology, Medical Faculty, Heinrich-Heine-University Düsseldorf, Düsseldorf, Germany; Department of Neurology, St. Josef-Hospital, Ruhr-University Bochum, Bochum, Germany; Department of Neurology, Justus-Liebig-University Giessen, Giessen, Germany; Department of Neurology, St. Josef-Hospital, Ruhr-University Bochum, Bochum, Germany; Department of Neurology, Hannover Medical School, Hanover, Germany; Department of Neurology, Justus-Liebig-University Giessen, Giessen, Germany; Department of Neurology, Medical Faculty, Heinrich-Heine-University Düsseldorf, Düsseldorf, Germany

**Keywords:** adverse effects, autoimmune comorbidity, B-cell depleting therapy, immunotherapy, psoriasis

## Abstract

**Background:**

B-cell depleting therapies (BCDT), including ocrelizumab, ofatumumab, and ublituximab, are highly effective disease-modifying therapies for multiple sclerosis (MS). Several case reports have raised concerns about new-onset or exacerbation of psoriasis under BCDT.

**Objectives:**

This article aims to analyze clinical characteristics, treatment courses, and outcomes of MS patients who developed or experienced worsening of psoriasis during BCDT.

**Design:**

This retrospective, multicenter analysis included patients from four German university hospitals (Düsseldorf, Hannover, Bochum, Giessen).

**Methods:**

We retrospectively screened 3228 MS patients under BCDT between 2020 and 2024 for development of psoriasis or an exacerbation of a known psoriasis. Clinical data, including Expanded Disability Status Scale, Psoriasis Area and Severity Index scores, treatment regimens, and comorbidities, were analyzed.

**Results:**

Among 3228 patients treated with BCDT, 7 developed new-onset psoriasis and 10 showed exacerbation of preexisting psoriasis. The median time to psoriasis onset or worsening was 13 months (3–83 months) under continuous treatment with BCDT. Topical therapies were effective in most cases, but a change of MS treatment or initiation of psoriasis-specific immunotherapies, including the interleukin-17A-antibody secukinumab, was required in four patients.

**Conclusion:**

Psoriasis onset or worsening during BCDT is rare. While most cases are manageable with standard psoriasis treatments, severe cases may necessitate therapy adjustments. The potential immunological interplay between MS and psoriasis treatment warrants further investigation.

## Plain language summary

Psoriasis in people with multiple sclerosis treated with B-cell therapies: an analysis from several medical centres

**Why was this study done?** Multiple sclerosis (MS) causes inflammation of the brain and spinal chord as well as a slowly progressing loss of function in daily life. Medicines called B-cell therapies are very effective in treating MS. However, there have been reports that some people develop the skin condition psoriasis, or see their psoriasis get worse, while on these treatments. We wanted to find out how often this happens, what it looks like in patients, and how it can be managed.

**What did the researchers do?** We looked back at the medical records of 3,228 people with MS who received B-cell therapy at four German hospitals between 2020 and 2024. We checked how many of them developed psoriasis for the first time or had their existing psoriasis get worse. We also collected information on the severity of psoriasis and MS as well as the treatments used.

**What did the researchers find?** Seventeen people developed psoriasis problems while on B-cell therapy. Seven patients developed psoriasis for the first time, and ten had worsening of psoriasis they already had. On average, this happened about one year after starting treatment. In most patients, standard creams and ointments worked well. In a few cases, however, doctors had to change the MS treatment or add a psoriasis-specific medicine.

**What do these results mean?** Psoriasis during B-cell therapy is uncommon. When it happens, it can often be treated with regular skin treatments, but sometimes stronger medicines or treatment changes are needed. More research is needed to understand why this happens and how MS and psoriasis treatments may affect each other.

## Introduction

Multiple sclerosis (MS) is the most common immune-mediated disease of the central nervous system and affects about 2.8 million people worldwide.^1^ Although MS cannot be cured, the landscape of disease-modifying therapies (DMT) has significantly broadened over the past 30 years. Anti-CD20 B-cell depleting therapies (BCDT) are particularly effective, even in patients with highly active disease, but require careful consideration of potential side effects such as infections or secondary autoimmune phenomena. Rituximab was first shown to be efficient in MS but has not been formally approved.^2^ Today, ocrelizumab, ofatumumab, and ublituximab are approved for MS treatment.^2,3^ Binding of these antibodies leads to complement- and antibody-dependent cytotoxicity, phagocytosis of B-cells, and CD20-positive T cells.^2,4^ BCDT lead to an almost complete depletion of CD20-positive B-cells in the peripheral blood and thereby influence the proinflammatory response of type 1 T helper (Th) and Th17 cells.^2^

Comorbid autoimmune-mediated diseases are frequently observed in patients with MS, with a particularly increased prevalence of psoriasis.^5,6^ This overlap can complicate the development of treatment regimens that address both conditions. Studies suggest that BCDT can induce or aggravate autoimmune comorbidities, especially those mediated by T cells such as inflammatory bowel disease or psoriasis.^7,8^

Case reports and adverse event database analyses on BCDT-associated psoriasis have highlighted the importance of understanding psoriasis as either a drug-induced or unmasked comorbidity in MS patients under BCDT.^8–14^ This leads to uncertainty regarding the use of high efficacy MS therapies in patients with prior skin diseases and therapeutic options for MS patients with comorbid psoriasis. This study aims to analyze the prevalence of BCDT-associated psoriasis exacerbations at four MS centers together with clinical characteristics of patients.

## Methods

### Study design and patients

Patients treated at the MS centers of the German university hospitals in Bochum, Düsseldorf, Giessen, and Hannover were retrospectively screened. Patients were included if they met the following criteria: (1) a diagnosis of MS according to the 2017 revision of the McDonald criteria^15^; (2) treatment with a BCDT between January 2020 and December 2024; and (3) either a new diagnosis of psoriasis after initiation of BCDT or documented worsening of preexisting psoriasis under BCDT. Patients with newly diagnosed psoriasis or worsening of preexisting psoriasis under BCDT were identified using International Classification of Diseases, Tenth Revision codes, complemented by manual review of available neurological and dermatological medical records. Patients with insufficient medical documentation following the first dose of BCDT were excluded. One case had been previously published.^14^

### Outcome measures

Medical history was obtained by reviewing the available documentation. MS and psoriasis activity and treatment history were analyzed retrospectively. Clinical activity was assessed with the Expanded Disability Status Scale (EDSS) for MS and Psoriasis Area and Severity Index (PASI). The PASI score indicates the affected percentage of skin surface and degree of skin erythema, induration, and scaling, resulting in a score ranging from 0 (no disease) to 72 (maximal disease severity). PASI scores were extracted from the available dermatological documentation. Patient age at MS diagnosis and BCDT initiation, prior MS treatments, and comorbidities were analyzed. Psoriasis treatment before exacerbation and changes to MS and psoriasis treatments after worsening or onset of psoriasis symptoms BCDT were assessed.

### Statistical analyses

Data were analyzed using Graph Pad Prism (Version 10.4.1; GraphPad Software, San Diego, CA, USA). Statistical analyses were descriptive; frequencies, means, standard deviations, medians, and ranges were calculated and presented in tabular form.

## Results

At the four participating MS centers, 3228 individual patients were treated with BCDT during the observation period. After initiation and continuation at the recommended treatment interval, seven patients developed a new psoriasis, while symptoms worsened in 10 out of 16 patients with a known psoriasis. The median time from BCDT initiation to onset of psoriasis exacerbation or new diagnosis was 13 months (range: 3–83).

These patients had a median age of 41.5 (range: 22–58) years at BCDT initiation. The cohort included nine male and eight female patients. Fourteen patients had relapsing-remitting MS (RRMS), two primary progressive (PPMS), and one secondary progressive MS (SPMS). The median EDSS at the time of BCDT initiation was 2.5 (range: 1.5–6.0). Thirteen patients received ocrelizumab, three ofatumumab, and one rituximab. Eight patients were treatment-naïve, eight had received platform therapies such as dimethyl fumarate (DMF), interferon, glatiramer acetate or teriflunomide, and one natalizumab before initiation of the BCDT.

### Psoriasis exacerbation during BCDT

Table 1 provides an overview of the 10 cases with exacerbation of a known psoriasis under BCDT. Six of these patients were treated with ocrelizumab, three with ofatumumab, and one with rituximab. Seven patients had prior treatment with lower efficacy therapies (DMF, interferon, or teriflunomide) and were switched to a BCDT due to clinical or radiological disease activity, while the rest were therapy naïve before the initiation of the BCDT. These patients had a median EDSS of 2.5 (range: 1.5–6.0) at the time of BCDT initiation, which remained stable 12 months after the start of the therapy. After initiation of the BCDT, psoriasis symptoms exacerbated from a median PASI of 0.9 (range: 0–3.9) up to a median PASI of 3.6 (range: 2–17) in this cohort. The median time from BCDT initiation to psoriasis exacerbation was 16.5 (range: 3–83) months.

**Table 1.** Summary of clinical and therapeutical features of patients with prior psoriasis that experienced an exacerbation under BCDT.

Case number	Case 1	Case 2	Case 3	Case 4	Case 5	Case 6	Case 7	Case 8	Case 9	Case 10
Age at initiation of BCDT	58	42	30	44	26	57	40	23	35	56
Sex	Female	Male	Female	Male	Female	Male	Male	Male	Female	Female
MS type	RRMS	SPMS	RRMS	RRMS	RRMS	RRMS	RRMS	RRMS	RRMS	RRMS
BCDT	OFA	RIT	OFA	OFA	OCR	OCR	OCR	OCR	OCR	OCR
Prior DMT	DMF	DMF, IFN	IFN, DMF	DMF, GLAT	None	None	DMF, IFN	DMF, IFN	Teriflunomide	None
Time from PsO diagnosis to start BCDT (years)	28	Unknown	4	5	2	4	12	9	24	25
Time from start BCDT to PsO exacerbation (months)	2	83	11	9	5	39	4	3	48	34
Psoriatic arthritis	Yes	Yes	No	No	No	Yes	No	No	No	No
PsO treatment before BCDT	None	Clobetasole* / DMSO nail polish*	None	None	None	Calcipotriol*	None	Calcipotriol*	None	Leflunomide
PsO treatment after exacerbation	Secukinumab	None	Calcipotriol*	Calcipotriol*	Calcipotriol*	Secukinumab	Calcipotriol*, PUVA*	Calcipotriol*	Cortico-steroids*	None
DMT after PsO exacerbation	None	Intrathecal triamcinolone	OFA	OCR	OCR	None	OCR	OCR	OCR	Cladribine

* topical treatment.

underlined: last DMT before BCDT.

BCDT, B-cell depleting therapies; DMF, dimethyl fumarate; DMSO, dimethyl sulfoxide; DMT, disease modifying therapy; GLA, glatiramer acetate; IFN, interferon; MS, multiple sclerosis; OCR, ocrelizumab; OFA, ofatumumab; PUVA, psoralen plus ultraviolet A; RIT, rituximab; RRMS, relapsing-remitting MS; SPMS, secondary progressive MS.

Not all these patients required psoriasis treatment prior to the start of the BCDT. After psoriasis worsening, seven patients started or modified psoriasis treatment, while three continued prior treatment with calcipotriol, topical clobetasol propionate, or remained without psoriasis-specific medication. One patient without prior psoriasis medication began treatment with topical corticosteroids. Four patients without psoriasis medication before the start of the BCDT began treatment with calcipotriol. Treatment was switched to secukinumab without any other MS-specific immunomodulatory therapy in one patient with prior treatment with calcipotriol (Case 6) and one patient without any psoriasis treatment at the time of the start of the BCDT (Case 1). Following therapeutic modifications, the 10 patients with a documented psoriasis exacerbation had a median PASI of 0.8 (range: 0–3).

### Stable preexisting psoriasis under BCDT

The participating clinics had also treated six patients with preexisting psoriasis who remained clinically stable without any psoriasis-specific therapy or with continued topical treatment after starting BCDT. One of these patients had received DMF, a therapy used for MS and psoriasis treatment, before switching to ocrelizumab.

### Psoriasis onset during BCDT

Table 2 summarizes the clinical features of seven patients without a prior psoriasis who developed the disease after initiation of a BCDT. All these patients were treated with ocrelizumab. This was the first MS-specific treatment for four of these patients, while one had received prior treatment with natalizumab and one with glatiramer acetate. At the start of the BCDT, these seven patients had a median EDSS of 3.7 (range: 2.0–5.5). After 12 months of treatment with BCDT the median EDSS was 4.0 (range: 2.0–6.0). Long-term data on MS disease activity after the therapeutic modifications in reaction to psoriasis symptoms are not yet available for all patients. The median time from BCDT initiation to psoriasis onset was 13 (range: 3–48) months. All these patients developed psoriatic skin lesions, while two patients also had psoriatic arthritis. The psoriasis symptoms progressed up to a median PASI of 10.2 (range: 3.7–26.0) in this subgroup. Three patients received calcipotriol as a psoriasis treatment, two patients were treated with a combination of topical therapy and oral glucocorticoids, and one patient was treated with secukinumab. One patient with known rheumatoid arthritis was treated with ocrelizumab in combination with methotrexate before the onset of psoriasis symptoms and received a combination of methotrexate, oral corticosteroid, and upadacitinib afterward (Case 13). After modification of psoriasis and MS treatments, the psoriasis symptoms improved to a median PASI of 1.95 (range: 0.7–6.8) in this subgroup.

**Table 2.** Clinical features of patients that developed new psoriasis under treatment with a BCDT.

Case number	Case 11	Case 12	Case 13	Case 14	Case 15		Case 16	Case 17
Age at initiation of BCDT	39	24	58	40		22	43	58
Sex	Male	Female	Female	Female		Male	Male	Male
MS type	PPMS	RRMS	RRMS	RRMS		RRMS	RRMS	PPMS
BCDT	OCR	OCR	OCR	OCR		OCR	OCR	OCR
Prior DMT	None	NTZ	None	GLAT, IFN		None	None	None
Time from start BCDT to first psoriasis symptoms (months)	13	3	10	10		31	16	48
Psoriatic arthritis	No	No	Yes	Yes		No	No	No
Psoriasis treatment	Calcipotriol*	Calcipotriol*	MTX, decortin, upadactinib	Secukinumab		Calcipotriol*, decortin	Calcipotriol*	Unspecified topical treatment*, prednisolone**
DMT after psoriasis onset	None	OCR	OCR	NTZ		OCR	OCR	OCR

* topical treatment.

** The patient was treated with oral prednisolone for concomitant sarcoidosis.

underlined: last DMT before BCDT.

BCDT, B-cell depleting therapies; DMT, disease modifying therapy; GLA, glatiramer acetate; IFN, interferon; MTX, methotrexate; NTZ, natalizumab; OCR, ocrelizumab; RRMS, relapsing-remitting MS.

### MS treatment changes in reaction to psoriasis

The psoriasis symptoms led to a change of the MS treatment regimen in 5 patients of all 17 analyzed cases. In two patients who started treatment with secukinumab, the BCDT was discontinued and no other MS-specific DMT was administered (Cases 1 and 6). One patient was switched from ocrelizumab to natalizumab and additionally received secukinumab (Case 14). Ocrelizumab treatment was discontinued in one patient with PPMS after he developed psoriasis, and the patient remained without any immunomodulatory MS treatment (Case 11). One RRMS patient’s treatment was changed from ocrelizumab to cladribine in reaction to the psoriasis exacerbation (Case 10). These treatment changes led to satisfactory psoriasis control in all cases. After the changes to the existing MS DMT and psoriasis treatment, the mean PASI of all analyzed patients improved from 8.2 (worst PASI after initiation of BCDT) to 1.6.

## Discussion

The management of psoriasis as an autoimmune comorbidity in patients with MS remains challenging in clinical practice. With the growing use of BCDT, concerns have emerged regarding a potential increase in the incidence or worsening of psoriasis under these treatments. In addition, the possibility of increased MS disease activity in response to certain substance groups used for treatment of psoriasis needs to be considered when choosing a treatment regimen in patients with these two comorbidities.^3,7^ Based on clinical data from four MS centers, our observations emphasize the importance of greater awareness of this comorbidity to guide more informed treatment decisions.

Reports of patients treated with rituximab for rheumatic or hemato-oncological diseases developing psoriasis were published even before BCDTs were approved for the treatment of MS.^16^ The first reported case of drug-induced psoriasis during ocrelizumab treatment was published in 2018, shortly after the drug was approved.^17^ Since then, several cases of psoriasis induced by ocrelizumab or rituximab treatment in patients with MS have been published,^9–13^ including one case included in this cohort.^14^ Table 3 provides an overview of previously published cases of BCDT-induced psoriasis or psoriasis exacerbations.

**Table 3.** Characteristics of published cases of psoriasis induced or exacerbated after initiation of BCDT.

Reference	BCDT	Age, sex	Prior psoriasis	Manifestation	MS type	Time to onset	Psoriasis treatment	MS treatment
Darwin et al.^17^	OCR	68, female	No	Skin	Not disclosed	4 months	Topical clobetasol	OCR continued
Molazadeh et al.^10^	RTX	39, female	No	Skin	RMS	4 cycles + 2 months	Topical steroids	RTX continued
Jakob Brecl et al.^11^	OCR	40, female	No	Skin	PPMS	6 months	Oral + topical treatment not specified	OCR discontinued
Jakob Brecl et al.^11^	OCR	66	No	Skin	PPMS	7 months	Topical	OCR discontinued
Lappi et al.^13^	OCR	68, female	No	Skin	Not disclosed	3.5 months	Topical clobetasol	Not disclosed
Emma et al.^12^	OCR	30, male	No	Skin	RMS	13 months	Topical steroids	OCR discontinued, diroximel fumarate planned
Esmaeili et al.^18^	OCR	34, female	No	Skin + arthritis	RMS	2 months	Secukinumab	OCR discontinued
Naranjo Guerrero et al.^9^	OCR	33, male	No	Nail	RMS	11 months	Clobetasol propionate nail lacquer	OCR continued
Naranjo Guerrero et al.^9^	OCR	36, male	No	Skin	RMS	4 months	Topical clobetasol propionate	OCR continued
Naranjo Guerrero et al.^9^	OCR	45, male	No	Skin	RMS	5 months	Topical calcipotriol/ betamethasone	OCR continued
Kölsche et al.^14^	OFA	58, female	Yes	Skin + arthritis	RMS	1 week	Secukinumab	OCR discontinued

BCDT, B-cell depleting therapies; MS, multiple sclerosis; OCR, ocrelizumab; OFA, ofatumumab; PPMS, primary progressive MS; RRMS, relapsing-remitting MS; RTX, rituximab.

**Figure 1.**
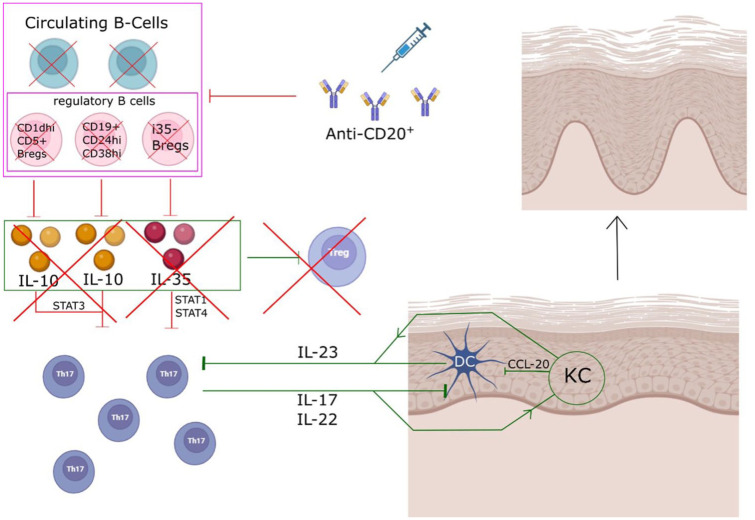
Psoriasis and B-cell depletion: exploring an immunological imbalance. B-cell-depleting antibodies in multiple sclerosis lead to the depletion of circulating B cells, including various subsets of regulatory B cells. Reduction of IL-10- and IL35-producing B_regs_ may result in reduced suppression of Th17 cells and impaired induction of regulatory T-cell differentiation.^22,24–26^ The remaining Th17 cells activate DCs and KCs via IL-17 and IL-22, establishing a feedback loop in which IL-23 production in the skin further promotes Th17 differentiation.^20^ Activated KCs also recruit additional DCs to the site of inflammation through CCL20.^20^ Source: Created by S. Faissner and U. Ceylan using BioRender.com and Inkscape. DC, dendritic cell; IL, interleukin; KC, keratinocyte; Th, T helper.

Psoriasis is a systemic inflammatory disease that primarily manifests as a skin disease in most patients but also causes psoriatic arthritis in 30% of cases.^19^ The pathophysiology of psoriasis is driven by a dysregulated cytokine release, such as interleukin (IL)-17, IL-23, tumor necrosis factor (TNF), and interferon gamma, from dendritic cells, Th1, and Th17 cells.^20^ In a mouse model, B cell depletion led to an increased psoriasiform inflammation in response to an external trigger, highlighting a possible role of CD20-positive cells in maintaining the homeostasis of inflammatory skin processes.^21^ Therefore, it could be conceivable that B cell-depletion leads to changes in B cell-mediated regulation of T cell function, resulting in increased pro-inflammatory processes in affected organs, including the skin.^22,23^ By transferring results from in vitro studies, Figure 1 provides an overview on possible cellular mechanisms by which BCDT may influence psoriasis pathology of the skin. Even without BCDT treatment, MS is associated with an increased risk for psoriasis^6^, which raises the question whether the reported cases of psoriasis are induced by a BCDT or solely associated with the underlying disease.

With a total of more than 3000 patients treated with a BCDT between January 2020 and December 2024 at all participating MS centers, a new psoriasis onset after treatment initiation was documented in only seven patients (0.2%) in this cohort. An exacerbation or new onset of psoriasis (*n* = 17) was documented in 0.49% of all patients treated with BCDT. Six patients with prior psoriasis diagnosis did not experience an exacerbation of psoriasis symptoms under BCDT. Since these data are derived from four MS centers in Germany, where patients with highly active MS or multiple comorbidities are typically treated, the findings suggest an overall low incidence of this phenomenon, which might even be overestimated considering the complex disease courses treated in specialized MS centers. Nevertheless, psoriatic or psoriasiform skin lesions under BCDT should prompt consideration of a therapy-related adverse event, although such occurrences remain infrequent.

In our analyzed cohort of 3228 MS patients treated with BCDT, we identified 23 patients with a psoriasis that was diagnosed either before or after the start of the BCDT. With 0.71%, the prevalence of psoriasis in this cohort was lower than reported psoriasis prevalence in the general population in Europe.^27^ This may be explained by the fact that BCDT are less likely to be prescribed in patients who receive psoriasis-specific immunotherapies to avoid drug interactions. Previous studies have yielded conflicting results regarding the association between psoriasis and BCDT in MS. Analysis of pharmacovigilance data reported a significantly increased number of psoriasis cases in patients treated with rituximab and ocrelizumab.^8^ In contrast, a single-center observational study found no evidence of psoriasis exacerbation among MS patients receiving BCDT.^28^ Notably, 5 of the 10 patients with exacerbation of preexisting psoriasis in our cohort had been treated with DMF prior to BCDT initiation, given its established efficacy in psoriasis, an effect of DMF discontinuation on subsequent psoriasis worsening cannot be excluded.

Patients in our case series developed new or worsening psoriasis symptoms a median 13 months after initiating BCDT, as we also included patients with a psoriasis worsening or onset more than 2 years after BCDT initiation. In previously published cases, symptom onset was typically reported within 6 months of treatment initiation.^9,10,13,16,17,29^ This temporal connection may suggest a causal relationship between BCDT and psoriasis.

In our cohort, patients with new-onset psoriasis had higher PASI scores compared with those experiencing exacerbation of preexisting disease. Patients with preexisting psoriasis experienced exacerbations up to a median PASI of 3.6, which is classified as mild, while a median PASI of 10.2 was documented in patients with new psoriasis onset. This may reflect earlier recognition and treatment in patients with known psoriasis, due to higher patient alertness and established access to dermatologic care, preventing skin lesions from progressing to higher PASI scores. Furthermore, patients with a higher psoriasis burden often require systemic immunotherapy, which may preclude the use of BCDT in addition to the psoriasis-specific therapy in patients with known highly active psoriasis. After therapeutic modifications psoriasis symptoms regressed to a PASI of 1.95 in patients with a new psoriasis diagnosis under BCDT and 0.8 in patients with preexisting psoriasis. These results are in line with the current therapeutic approach to psoriasis, which follows a treat-to-target strategy aiming for PASI 90, defined as a 90% reduction from baseline, or an absolute PASI score of <2.^30^

### Treatment strategies

Mild psoriasis of the skin is often treated with topical therapies such as calcipotriol or corticosteroids. Traditional systemic therapies include methotrexate, ciclosporine A, DMF, or a phosphodiesterase-4 inhibitor.^20^ In recent years several biologicals, such as TNF-α inhibitors, anti-IL17 and anti-IL23 antibodies and Janus kinase and Tyk2 inhibitors have also been approved for treatment of moderate or severe psoriasis and psoriatic arthritis.^31^ In some of the previously published cases of BCDT-induced psoriasis, topical corticosteroid therapy led to a sufficient improvement of psoriasis symptoms so that no change to the MS therapy was needed.^9,10,13,17^ Accordingly, in most of the cases reported here, basic psoriasis treatment was sufficient to control the dermatological symptoms; hence, no discontinuation of the BCDT was needed. This proves that adequate psoriasis treatment should first be established and a discontinuation of the BCDT only be considered once the psoriasis-specific therapy with oral or topical corticosteroids as well as topical calcipotriol cannot sufficiently control the symptoms.

The presented case series includes one PPMS patient in which the psoriasis onset led to a discontinuation of ocrelizumab and no other MS-specific DMT was started due to the limited therapeutic options for the treatment of this MS form. Analogically, one previously published case report describes two patients with PPMS that developed psoriasis after initiation of ocrelizumab in which the psoriasis was treated with oral and topical therapy and ocrelizumab discontinued.^11^ With studies on Bruton tyrosine kinase inhibitors for the treatment of progressive MS forms and the possibility of the approval of the first substance in the near term, new treatment options may soon become available.

Cases of psoriasis onset or worsening during BCDT in which treatment with classical oral or topical psoriasis therapies do not lead to sufficient control of symptoms present a greater difficulty. DMF is approved for the treatment of MS and psoriasis and is thus a valid therapeutic option for patients with these two comorbidities.^7,28^ Accordingly, five of the presented cases with a known psoriasis had been treated with DMF before initiation of the BCDT. Sphingosine-1-phosphate receptor (S1PR) modulators, which inhibit lymphocyte migration from lymph nodes, are approved for the treatment of MS and have also demonstrated clinical benefits for psoriasis patients, as measured by PASI.^32^ Although not approved for the treatment of psoriasis, S1P receptor modulators represent a valuable therapeutic option for patients with both MS and psoriasis.^7,22,32^ However, MS patients with prior treatment with BCDT often require higher efficient treatment than DMF or S1PR-modulators to suppress MS disease activity, which explains why these therapeutic options were not implemented in the cases presented above.

Moderate and severe forms of psoriasis often require systemic treatment with biologicals, of which the systemic immunological effects and interactions with other immunotherapies are not completely understood. We previously published one of the cases included in the presented case series, in which a patient experienced an exacerbation of preexisting psoriasis under ofatumumab therapy and was successfully treated with the anti-IL17A antibody secukinumab without an MS-specific DMT.^14^ This case series now includes a second MS patient who was switched from ocrelizumab to secukinumab after a psoriasis exacerbation, a therapeutic pathway that has been previously reported in another case report.^18^ A proof-of-concept study comparing secukinumab to placebo in patients with relapsing MS demonstrated a trend toward disease stabilization on MRI, as evidenced by a significant reduction in the cumulative number of new gadolinium-enhancing T1 lesions.^33^ However, the study did not meet its primary endpoint: compared with placebo, secukinumab did not significantly reduce the number of combined unique active lesions observed on monthly MRI scans from weeks 4 to 24. Secukinumab has thus been suggested to be a safe psoriasis treatment option in patients with comorbid psoriasis and may prevent inflammation in mildly active MS.^7,34^

In some patients with high MS inflammatory activity, treatment with secukinumab without an MS-specific therapy alone is insufficient to control MS disease activity. A published case describes a patient with both psoriasis and MS, in whom secukinumab alone failed to adequately control MS disease activity, while rituximab treatment worsened psoriasis symptoms.^35^ In such cases, a combination therapy with two immunomodulatory therapeutics might be necessary. A recent multi-center study analyzed the data of patients with MS that were treated with anti-IL17A antibodies for concomitant rheumatological diseases.^36^ In that cohort, four of the total six patients that were treated with anti-IL17A monotherapy had new MS disease activity, while no relevant safety concerns occurred in the seven patients receiving additional MS-specific DMTs.^36^ As part of this case series, we present a patient who was successfully treated with a combination of natalizumab and secukinumab. Singular case reports have also been published on cases with psoriasis onset or exacerbation after initiation of natalizumab, but so far, no data have been published to suggest a causative link.^37^

Out of the presented 17 cases with BCDT-induced psoriasis, 5 patients experienced psoriatic arthritis, which is consistent with the prevalence of arthritis manifestation in psoriasis of the general population.^19^ In our cohort, all three patients treated with secukinumab had experienced joint involvement before. Topical treatment of skin manifestations alone is insufficient for patients with psoriatic arthritis, who often require systemic immunotherapy. Thus, early consideration of systemic psoriasis therapy and modification of MS treatment may be warranted in patients presenting with psoriatic arthritis.

### Limitations

This study has several limitations. Its retrospective design relies on the accuracy and completeness of medical records and limits the assessment of the causal relationship between BCDT and psoriasis symptoms. Psoriasis symptoms may have been underreported by patients or underdiagnosed, particularly in cases with mild manifestations, possibly influencing the calculation of a psoriasis prevalence in this cohort. Furthermore, differences in prior DMT, including varying half-lives and washout strategies before initiation of BCDT, may have confounded analyses of time to psoriasis onset or exacerbation and could not be systematically accounted for in this retrospective study. Due to the retrospective study design, quality-of-life data were not available, although psoriasis symptoms often substantially impair patients’ quality of life and thereby influence treatment decisions.

## Conclusion

New onset or worsening of preexisting psoriasis under BCDT is rare, occurring in only about 0.5% of cases. However, it should still be considered, especially since it is generally well treatable with oral or topical therapies without BCDT discontinuation. In patients with a high psoriasis burden or psoriatic arthritis, discontinuation of BCDT can be necessary and IL-17A blockade with secukinumab may offer effective disease control and represents a suitable option, particularly in MS patients with low disease activity, as it may positively influence both conditions. In cases of high MS disease activity, combined treatment with an MS-specific DMT and systemic psoriasis therapy may be necessary, although safety data on such combinations remain limited.

## Appendix

### Abbreviations

BCDT B-cell depleting therapies

DMF dimethyl fumarate

DMT disease modifying therapies

EDSS Expanded Disability Status Scale

GLA glatiramer acetate

IFN interferon

IL interleukin

MS multiple sclerosis

MTX methotrexate

NTZ natalizumab

OCR ocrelizumab

OFA ofatumumab

PASI psoriasis area and severity index

PPMS primary progressive multiple sclerosis

PUVA psoralen plus ultraviolet A

RIT rituximab

RRMS relapsing-remitting multiple sclerosis

SPMS secondary progressive MS

Th T helper

TNF tumor necrosis factor

## Declarations

**Ethics approval and consent to participate:** The Ethics Committee of Heinrich Heine University granted approval for the study (2016-2455_2). An individual, written informed consent was not necessary due to the study design (retrospective analysis of data collected during routine clinical practice). The original ethical approval is from 2016 (No. 2016-2455_2), but there is also an amendment from 09.12.2021 (Study No. 5794R) in which the Ethics Committee of Heinrich Heine University approved the prospective collection and retrospective analysis of the anonymized patient data. According to the initial approval and amendment further specific ethical approval for this specific data analysis is not necessary when using anonymized data.

**Consent for publication:** See above, not applicable.

**Availability of data and materials:** Key data are already presented in two tables in the article. The raw data are not publicly available, as they contain identifiable patient-related information. Reasonable requests for access to de-identified data may be considered by the corresponding author.
